# The efficacy and safety of Hirudin plus Aspirin versus Warfarin in the secondary prevention of Cardioembolic Stroke due to Nonvalvular Atrial Fibrillation: A multicenter prospective cohort study

**DOI:** 10.7150/ijms.52752

**Published:** 2021-01-09

**Authors:** Chang-geng Song, Li-jie Bi, Jing-jing Zhao, Xuan Wang, Wen Li, Fang Yang, Wen Jiang

**Affiliations:** Department of Neurology, Xijing Hospital, Fourth Military Medical University, Xi'an, China.

**Keywords:** Hirudin, aspirin, warfarin, secondary prevention, cardioembolic stroke, nonvalvular atrial fibrillation

## Abstract

**Background:** To investigate the efficacy and safety of hirudin plus aspirin therapy compared with warfarin in the secondary prevention of cardioembolic stroke due to nonvalvular atrial fibrillation (NVAF).

**Methods:** Patients with cardioembolic stroke due to NVAF were prospectively enrolled from 18 collaborating hospitals from Dec 2011 to June 2015. Fourteen days after stroke onset, eligible patients were assigned to the hirudin plus aspirin group (natural hirudin prescribed as the traditional Chinese medicine Maixuekang capsule, 0.75 g, three times daily, combined with aspirin 100 mg, once daily) or the warfarin group (dose-adjusted warfarin targeting international normalized ratio (INR) 2-3, with an initial daily dose of 1.25 mg). Patients were followed up at 1, 2, 3, 6, 9, and 12 months after stroke onset. Time in therapeutic range (TTR) was calculated according to Rosendaal methodology to evaluate the quality of INR management in the warfarin group. The primary efficacy endpoint was the recurrence of stroke within 12 months after stroke onset. Safety was assessed as the occurrence of the composite event “intracranial hemorrhage and other bleeding events, death, and other serious adverse events”. The Cox proportional hazard model and Kaplan-Meier curve were used to analyze the efficacy and safety events.

**Results:** A total of 221 patients entered final analysis with 112 patients in the hirudin plus aspirin group and 109 in the warfarin group. Over the whole duration of our study, TTR for patients taking warfarin was 66.5 % ± 21.5%. A significant difference was not observed in the recurrence of stroke between the two groups (3.57% vs. 2.75%; *P* = 0.728). The occurrence of safety events was significantly lower in the hirudin plus aspirin group (2.68% vs.10.09%; *P* = 0.024). The risk for efficacy event was similar between the two groups (hazard ratio (HR), 1.30; 95% confidence interval (CI), 0.29-5.80). The safety risk was significantly lower in the hirudin plus aspirin group (HR, 0.27; 95% CI, 0.07-0.95). Kaplan-Meier analysis revealed significant difference in the temporal distribution in safety events (*P* = 0.023) but not in stroke recurrence (*P* = 0.726).

**Conclusion:** Significant difference in efficacy was not detected between warfarin group and hirudin plus aspirin group. Compared with warfarin, hirudin plus aspirin therapy had lower safety risk in the secondary prevention of cardioembolic stroke due to NVAF.

## Introduction

Ischemic stroke remains the most common type of stroke, affecting more than 11 million people worldwide each year 1, 2. Cardioembolic stroke accounts for approximately 20% of ischemic strokes 3, and its proportion in patients is increasing.^4^ With nonvalvular atrial fibrillation (NVAF) as the most leading cause 5, cardioembolic stroke is characterized by the sudden onset of neurological deficits, the occlusion of distal arteries supplying the cerebral cortex, and the resultant lesions in cortical territory 6. Moreover, cardioembolic stroke is associated with the high risk of recurrence 7 and long-term disability or mortality 8. Therefore the prophylaxis of NVAF-induced cardioembolic stroke and its recurrence is of extreme importance.

Warfarin has been used as the mainstay anticoagulant in the secondary prevention of cardioembolic stroke 6, 9, and its efficacy has been verified in several randomized trials 10-12. However, several limitations of warfarin have greatly impeded its use, such as the risk of bleeding 13, the requirement for frequent monitoring of international normalized ratio (INR) 14, and multiple interactions with various pharmaceuticals 15. In fact, the underuse of warfarin in the secondary prevention of cardioembolic stroke due to NVAF is a prevalent problem worldwide 16, and this problem is especially salient in China 17, 18. New oral anticoagulants (NOACs) do not require INR monitoring, and they are expected to be reasonable alternatives to warfarin 19. However, their higher price makes them very difficult to be widely used in China 18, 20. Therefore, it is urgent to find a solution to the dilemma of anticoagulant underuse in China, and traditional Chinese medicine could be a complementary option.

As the dominant active ingredient of the traditional Chinese medicine Maixuekang capsule, hirudin is a potent and selective thrombin inhibitor extracted from the peripharyngeal glands of medicinal leeches 21, 22. Maixuekang capsule, prepared from *Hirudo nipponica Whitman*, is an accessible and affordable traditional Chinese medicine in China. The therapeutically anticoagulant effects of natural hirudin and its analogs, lepirudin, desirudin, and bivalirudin have been verified in heparin-induced thrombocytopenia, deep vein thrombosis, and acute coronary syndromes 23-26. However, it remains to be studied whether natural hirudin could be used in the prevention of cardioembolic stroke.

According to current guidelines on secondary prevention of stroke 19, 27, 28, aspirin and aspirin combined with clopidogrel provide alternatives for patients in whom warfarin is inapplicable. However, aspirin alone is significantly less efficacious than warfarin 10, 29, 30, and aspirin plus clopidogrel has also been verified to be inferior to warfarin in both efficacy and safety 31. In this study, in the aim to find a possible solution to underuse of warfarin in China, we examined the efficacy and safety of hirudin plus aspirin combination therapy and compared them with that of warfarin in the secondary prevention of cardioembolic stroke due to NVAF.

## Materials and Methods

### Design and setting

This study was a multicenter prospective cohort study with Xijing Hospital as sponsor and other 17 collaborating hospitals in Shaanxi Province, northwest China. This study was approved by the ethics committees of Xijing Hospital (KY20140415-5), and complete approval was obtained from ethics committees in other participating hospitals. Written informed consent was obtained from all the patients or the guardians of the patients. All procedures in this study were in accordance with the Declaration of Helsinki and the International Conference of Harmonization Guidelines for Good Clinical Practice. This study was registered on ClinicalTrials.gov (NCT02181361).

### Patients

From December 2011 to June 2015, patients with cardioembolic stroke due to NVAF from 18 collaborating hospitals were enrolled in this study. The inclusion criteria were as follows: (1) age of 18 years or older with NVAF-related cardioembolic stroke; (2) diagnosis of cardioembolic stroke conformed to the TOAST (Trial of Org 10172 in Acute Stroke Treatment) classification system in terms of the typical clinical presentation, neuroimaging profile, and vascular and cardiac evaluation 32; (3) patients who were beyond 14 days of stroke onset and with stable clinical status. Patients were excluded if they met any of the following criteria: (1) patients with rheumatic heart disease or history of heart valve surgery; (2) patients with acute coronary syndrome or percutaneous coronary intervention within 30 days previous to enrollment; (3) patients with active infective endocarditis; (4) patients with purpura disease or blood coagulation disorder; (5) patients who had active bleeding or the tendency to bleed; (6) patients with history of intracranial hemorrhage (ICH) or other serious bleeding events; (7) patients diagnosed with peptic ulcer disease within 30 days previous to enrollment; (8) patients who had esophageal varices; (9) patients who had trauma or major surgery within 30 days previous to enrollment or patients who planned to have major surgery; (10) patients with persistent blood pressure of 180/100 mmHg or greater with or without anti-hypertension treatment; (11) patients who needed chronic anticoagulant treatment due to disorders other than AF; (12) patients with severe liver and kidney dysfunction; (13) patients who were allergic to warfarin, aspirin, or hirudin; (14) female patients who were pregnant or lactating.

### Baseline assessment

At the time of enrollment, demographics, medical histories including stroke, coronary heart disease, hypertension, diabetes mellitus, hyperlipidemia, cigarette smoking, and drinking, and the application of anticoagulants before enrollment were recorded. Physical examination was carried out, and clinical scores were evaluated for each patient, including Glasgow Coma Scale (GCS), National Institute of Health Stroke Scale (NIHSS), CHA2DS2-VASc (congestive heart failure, hypertension, age ≥ 75 [doubled], diabetes, stroke [doubled], vascular disease, age 65-74, and sex category [female]), and HAS-BLED (hypertension, abnormal renal/liver function [1 point each], stroke, bleeding history or predisposition, labile international normalized ratio [INR], elderly [>65], drugs/alcohol concomitantly [1 point each)]). Labile INR in HAS-BLED was not included due to unavailable information. Routine laboratory tests for the enrolled patients were conducted (blood routine, stool routine, serum lipid, coagulation function). Levels of platelets and coagulation indices, including prothrombin time (PT), thrombin time (TT), activated partial thromboplastin time (APTT) and international normalized ratio (INR), were recorded.

### Treatment

In the hirudin plus aspirin group, patients were prescribed with natural hirudin 0.75 g, three times a day (brand name: Maixuekang capsule; Guizhou Xinbang Pharmaceutical Co., China; Authorized Document Number: Z20033197 in Chinese medicine by the National Medical Products Administration of China; each capsule [0.25 g] contains an amount of anticoagulant equal to 11.2-16.8 antithrombin units [ATU]; one ATU equals the amount of anticoagulant that neutralizes 1 international unit (IU) of thrombin 21) and aspirin 100 mg, once daily 33. This dosage of Maixuekang capsule was chosen based on the drug instructions provided by manufacturer, relative provisions in the *Chinese Pharmacopoeia* 34, and previous studies 35, 36. For the warfarin group, patients were initially prescribed with a daily dose of 1.25 mg of warfarin. Three days later, INR of patients was checked every three days and the dose of warfarin was adjusted until it reached the target range of 2 to 3. Since then, INR monitoring was performed at each follow-up of 1, 2, 3, 6, 9, and 12 months after stroke onset, targeting an INR between 2 and 3, and the dose of warfarin was adjusted accordingly. Other medical interventions on ischemic stroke adhered to the guidelines on management and secondary prevention of stroke 28, 37. To check and ensure the adherence of medication, at each follow-up, patients in both warfarin group and hirudin plus aspirin group were queried by their supervising physicians to self-report their adherence to medication. We did this inquiry in a blame-free and non-judgmental way as much as possible, to make sure the patients feel comfortable and welcomed to share their adherence state. In addition, patients were required to bring the empty blister packs at each follow-up and the supervising physicians would check them to further confirm the medication adherence. Moreover, during the whole study period, the supervising physicians would contact their patients about every 14 days to check the well-being and medication adherence via phone call, mobile telephone text message and Wechat (instant messaging software, Tencent, Shenzhen, China). These methods helped us to evaluate and improve the medication adherence. In case of unwanted bleeding caused by hirudin overdose, we prepared prothrombin complex concentrate and desmopressin at each collaborating center. These two agents were reported to be effective to treat hirudin-induced bleeding 38, 39. We also had hemodialysis system at each collaborating center, which could rapidly remove hirudin from circulation 40, 41.

### Outcome measurement

The primary efficacy endpoint was defined as the recurrence of cardioembolic stroke due to NVAF within 12 months after stroke onset. Safety was evaluated as occurrence of the composite event “ICH and other bleeding events, death, and other serious adverse events” within 12 months after stroke onset. Patients were followed up at 1, 2, 3, 6, 9, and 12 months after stroke onset in a face-to-face interview by neurologists in every collaborating hospital. When all efforts to reach patients had been made but failed, these patients were recorded as those lost in follow-up. In each follow-up, the occurrence of efficacy and safety events was recorded. Routine laboratory tests were carried out to detect the levels of platelets, PT, TT, APTT and INR.

In order to evaluate the quality of INR management in the warfarin group, time in therapeutic range (TTR) was calculated according to Rosendaal methodology 42. This methodology assumes that INR values vary linearly between two consecutive measurements and is widely accepted for calculating individual patient-based percentage time in range 42-47. In addition, the proportion of patients achieving INR stability was also calculated. INR stability was defined as minimum of six months within the target INR range of 2.0-3.0 48.

### Statistical analysis

Continuous variables fitting the normal distribution were expressed as mean ± standard deviation (SD) and compared using Student's t-test when necessary, while skewed data were expressed as median (interquartile range [IQR]) and were compared using Mann-Whitney U test. Chi-squared test was used for categorical variables. To compare the risk of both efficacy and safety events for warfarin and hirudin plus aspirin treatment, hazard ratio (HR), which could be interpreted as relative risk, was obtained by the Cox proportional hazard model 49. The Kaplan-Meier curve with log-rank test was used for graphical assessment of time-dependent events 50. Logistic regression was used to detect the associated factors for both efficacy and safety events. Demographic features (age and sex), baseline clinical characteristics, and treatment regimen were investigated by univariate regression analysis, followed by multivariate regression which included variables with statistical significance from the univariate regression after adjusted for age and sex. Statistical analyses were performed using SPSS 25.0 (IBM Inc., Chicago, IL, USA). Two-tailed *P* < 0.05 was considered statistically significant.

## Results

**Figure 1:**
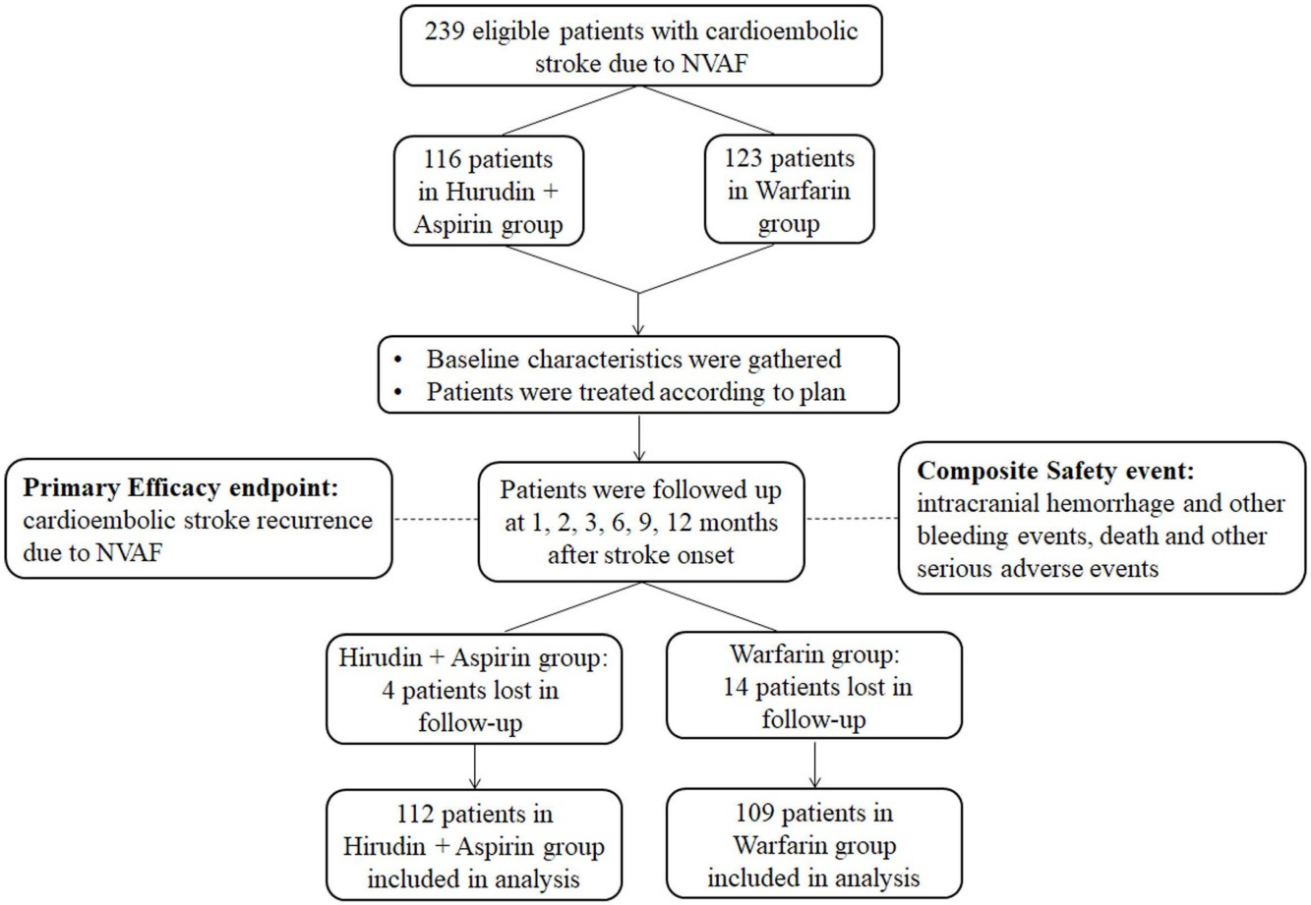
Flowchart of the study. Abbreviation: NVAF: nonvalvular atrial fibrillation.

From December 2011 to June 2015, a total of 239 eligible patients of cardioembolic stroke due to NVAF were enrolled in this study. One hundred and sixteen patients received hirudin plus aspirin treatment, while the other 123 received warfarin treatment. Four patients and 14 patients were lost to follow-up in the hirudin plus aspirin group and the warfarin group, respectively. A total of 112 patients in the hirudin plus aspirin group and 109 patients in the warfarin group entered final analysis (Figure 1).

### Baseline characteristics

Baseline characteristics of the hirudin plus aspirin and warfarin groups were demonstrated in Table 1. There were no significant differences in age, sex, GCS score, NIHSS score, CHA2DS2-VASc, and HAS-BLED between the two groups. Hypertension was found in 51.1% (113/221) of the patients, 41.2% (91/221) of the patients had coronary heart disease, and 29.9% (66/221) of the patients had previous history of stroke. Approximately one-third of patients in both groups had been prescribed with anticoagulants previously. There were no significant differences in level of platelets and coagulation indices, including PT, TT, APTT, and INR between the two groups.

### The relative risks for clinical outcomes

Patients in both groups were followed up at 1, 2, 3, 6, 9, and 12 months after stroke onset to evaluate the occurrence of primary efficacy endpoint and safety endpoint. The value of platelets PT, TT, APTT, and INR in each follow-up is presented in Figure S1. The INR in the warfarin group was adjusted to target the range between 2-3, with median value of 2.00, 1.98, 2.20, 2.34, 1.97, and 2.42 in the six follow-ups. Over the whole duration of our study, TTR for patients taking warfarin was 66.5% ± 21.5%, which means patients' spent 66.5 % of the total treatment duration within the therapeutic range. Patients taking warfarin spent 31.4% ± 19.3% of the total treatment duration with INR < 2. Only one patient spent time with INR > 3 for 1.3 months. This patient had non-fatal intracranial hemorrhage at 3 months after stroke onset and the follow-up was stopped. The proportion of patients with different TTR is shown in Table 2. Approximately half of the patients (49.5%) spent over 70% of the treatment duration within the therapeutic range. 77.9% patients achieved INR stability.

The occurrence of efficacy and safety events of the whole study is summarized in Table 3. The total number of both efficacy and safety events in hirudin plus aspirin group and warfarin group was 7 versus 14 respectively (6.25% vs. 12.84%; *P* = 0.095, Chi-squared test). At the end of the study, there is no significant difference in the recurrence of stroke between hirudin plus aspirin group and warfarin group (3.57% vs. 2.75%; *P* = 0.728, Chi-squared test). The occurrence of the composite safety events in the hirudin plus aspirin group is significantly lower than that of the warfarin group (2.68% vs. 10.09%; *P* = 0.024, Chi-squared test). The three safety events that occurred in the hirudin plus aspirin group were ecchymosis, hemorrhinia, and hematuria. Of the 11 safety events in the warfarin group, there were four cases of ecchymosis, four cases of hemorrhinia, one case of non-fatal ICH, one case of hematuria, and one case of hematochezia. Results from the Cox proportional hazard model revealed no significant difference in relative risk for the recurrence of stroke between the hirudin plus aspirin group and the warfarin group (HR, 1.30; 95% confidence interval (CI), 0.29-5.80). The risk for the occurrence of safety events was significantly lower in the hirudin plus aspirin group than in the warfarin group (HR, 0.27; 95% CI, 0.07-0.95).

### The occurrence of outcomes over time

**Figure 2:**
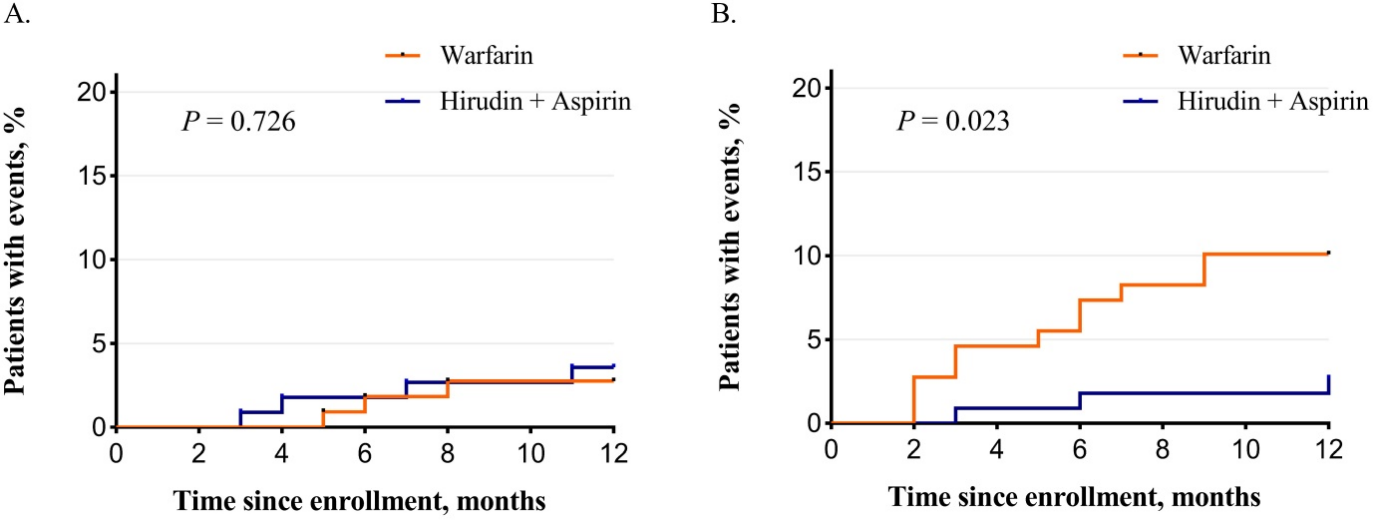
Kaplan-Meier curves for the primary efficacy endpoint and composite safety event. (A) Primary efficacy endpoint was the recurrence of stroke. There was no significant difference between hirudin + aspirin group and warfarin group (P = 0.726). (B) Composite safety event was intracranial hemorrhage and other bleeding events, death and other serious adverse events. The incidence was significantly lower in the hirudin + aspirin group (P = 0.023).

The occurrence of primary efficacy endpoint and safety outcomes occurring over time is graphically demonstrated in Figure 2. Kaplan-Meier analysis revealed that the incidence of safety events over time was significantly lower in the hirudin plus aspirin group than in the warfarin group (Log-rank test, *P* = 0.023, Figure 2B), but significant difference was not detected in efficacy events between the two groups (Log-rank test, *P* = 0.726, Figure 2A).

### Results of logistic regression analysis

The results of the logistic regression analysis for the efficacy events are presented in Table 4. Univariate regression analysis revealed that there was a significant difference regarding drinking between patients who had stroke recurrence and those who didn't (OR 5.007, 95% CI 1.081-23.186, *P* = 0.039). After being adjusted for age and sex in multivariate analysis, drinking was found to be positively associated with the happening of stroke recurrence (OR 5.007, 95% CI 1.081-23.186, *P* = 0.039).

The results of the logistic regression analysis for the incidence of composite safety events are summarized in Table 5. Univariate regression analysis showed that there was significant difference regarding age (OR 0.922, 95% CI 0.873-0.973, *P* = 0.003), hirudin plus aspirin therapy (OR 0.245, 95% CI 0.066-0.905, *P* = 0.035), HAS-BLED (OR 0.568, 95% CI 0.361-0.894, *P* = 0.014), previous anticoagulant use (OR 4.022, 95% CI 1.296-12.478,* P* = 0.016), and baseline PT level (OR 0.483, 95% CI 0.236-0.989, *P* = 0.046) between patients who had safety events and those who didn't. Multivariate logistic regression analysis showed that age (OR 0.897, 95% CI 0.873-0.960, *P* = 0.002), hirudin plus aspirin therapy (OR 0.217, 95% CI 0.051-0.925, *P* = 0.039), previous anticoagulant use (OR 6.355, 95% CI 1.690-23.892, *P* = 0.006), and baseline PT level (OR 0.328, 95% CI 0.136-0.789, *P* = 0.013) was independently associated with the occurrence of composite safety events. Among them, age, the choice of hirudin plus aspirin therapy, and baseline PT level were inversely associated with the occurrence of safety events while previous use of anticoagulant was positively related to the occurrence of safety events.

## Discussion

Conducted in the real-world clinical environment, our study made an exploratory investigation to test the efficacy and safety of hirudin plus aspirin combination therapy in the secondary prevention of cardioembolic stroke due to NVAF. While significant difference was not detected in the efficacy to prevent stroke recurrence between warfarin and hirudin plus aspirin therapy, the combination therapy could significantly reduce the risk of bleeding. These results provide preliminary evidence that, for patients who are unable to take warfarin and who can't afford NOACs, the combination of hirudin plus aspirin could be a reasonable candidate to substitute warfarin in the secondary prevention of cardioembolic stroke due to NVAF.

The underuse of warfarin in AF patients with ischemic stroke is a global problem 16, and this problem is particularly significant in China 20. Among ischemic stroke patients with NVAF, the rate of being discharged with warfarin was reported to be as low as 19.4% in China 17, 18, which is remarkably lower than that of approximately 40% in Europe 51 and at least 60% in the US 52, 53. Apart from the underuse of warfarin, even in patients who have received warfarin, INR monitoring was not performed well. In a Chinese real-world study in which 96 patients of NVAF induced ischemic stroke were prescribed with warfarin, INRs of 55.2% patients were below the therapeutic window of 2-3, and INRs of 41.7% patients were missing 54. In our study, we did our utmost to adjust the dose of warfarin to keep the INR within the range of 2-3. The mean TTR of 66.5 % in our study is comparable to previous studies 43, 45-47. TTR over 70% was reported to indicate excellent level of INR control 43, 47. In our study, 49.5% of patients taking warfarin achieved TTR over 70%. The proportion of 49.5% is also comparable to previous studies 43 and even better than some studies 45, 47. INR stability was associated with decreased thromboembolic events and bleedings and prolonged survival in NVAF 48. In our study, INR stability was achieved in 77.9% of patients taking warfarin, which is also higher than previous study 48. Taken together, these results suggested that our study had a relative satisfactory INR management.

Several underlying reasons for the underuse of warfarin in China have been identified. Warfarin possesses several inherent limitations, including the variation of efficacy among individuals, the multiple interaction with other pharmaceuticals, the request for frequent INR monitoring and risk of bleeding 14, 55-57. For the neurologists, apart from the concern of bleeding risks, the main barrier preventing them from prescribing warfarin to patients without contraindications is the lack of access to coordinated regular INR testing, rather than their knowledge gap or attitude 18. For the patients, the fear of bleeding risk and the trouble of frequent INR monitoring lead to their refusal to take warfarin 18. Socioeconomically, inadequate community-based health service and the lack of availability of INR self-monitoring devices increase the difficulty of frequent INR monitoring in China. New oral anticoagulants (NOACs) such as dabigatran, rivaroxaban, and apixaban, do not need INR monitoring and are efficacious in preventing stroke recurrence without increasing bleeding risks 58-60. However, their higher price hinders their generalized use. The status quo of warfarin underuse calls for alternative efficacious, safe, and convenient therapy in the secondary prevention method. Both the natural hirudin (Maixuekang capsule) and aspirin used in our study are affordable and available drugs in China. The improved safety of hirudin plus aspirin therapy, combined with its non-significant difference in efficacy compared with dose-adjusted warfarin, suggests that hirudin plus aspirin therapy has the potential to be a rational substitute for warfarin in the secondary prevention of NVAF-induced cardioembolic stroke, and therefore a likely solution to the underuse of warfarin in China. However, we should notice that in this study, the incidence of efficacy event is relatively low. This meant that we should be extremely cautious when we interpret the results concerning the effectiveness. Instead of announcing that the two arms has similar efficacy, it's more reasonable to conclude that so far we didn't detect significant difference in the efficacy.

Hirudin, isolated from the peripharyngeal glands of medicinal leeches, is a peptide of 65 amino acids and a selective and potent thrombin inhibitor 14 21. Hirudin inactivates thrombin by forming a tight and irreversible binding complex in a 1:1 stoichiometric manner and with a dissociation constant of 10-14 mol/l 61, 62. The equimolar hirudin-thrombin complex is stable throughout the physiological range of pH, and all the biological functions of thrombin are blocked, such as fibrinogen clotting, the activation of clotting factors V, VIII, and XIII, and the thrombin-induced platelet activation 21. In our study, natural hirudin was prescribed in the form of Maixuekang capsule. Maixuekang capsule is a traditional Chinese medicine prepared from the lyophilized powder of *Hirudo nipponica Whitman*, whose dominant anticoagulant component is hirudin 63, 64. Maixuekang capsule is economically more affordable than NOACs and more convenient to comply with than warfarin. Previous studies have reported that Maixuekang capsule could exert anticoagulant effects in lower extremity deep venous thrombosis (DVT) 65, facilitate long-term functional recovery for patients with acute ischemic stroke when combined with human urinary kallidinogenase 36, and improve the cognitive function in Alzheimer's disease (3g/day) when combined with donepezil 63. Our study found that when combined with aspirin, Maixuekang capsule demonstrated no significant difference in the efficacy to prevent the recurrence of cardioembolic stroke due to NVAF and lower bleeding risk when compared with warfarin. This finding extends our understanding of the utilization spectrum of Maixuekang capsule and natural hirudin.

Risk factors for stroke recurrence differ among different subtypes of index stroke. Previous study demonstrated that in cardioembolic stroke, older age was a predictor for stroke recurrence 66. In our study, multivariate logistic regression didn't detect the association between age and stroke recurrence. This could be due to the narrow age distribution of our cohort (69.45 ± 9.57 years for the whole cohort, data presented as mean ± SD), which provided insufficient stratification to detect difference in stroke recurrence between young and old patients. In our study, multivariate regression analysis found that drinking was positively associated with the recurrence of stroke. This result is consistent with previous findings that alcohol intake increased the risk of stroke 67, 68. Based on the present results, we recommend that NVAF patients with initial cardioembolic stroke quit drinking in their secondary prevention process. As for the incidence of safety events, multivariate logistic regression revealed that hirudin plus aspirin therapy was inversely associated. This suggested that in the real-world clinical practice in our study, hirudin plus aspirin therapy could reduce the risk of safety events compared with warfarin. Moreover, multivariate regression analysis also revealed that age and baseline PT levels were inversely related to the occurrence of safety events. However, previous studies reported that advanced age was positively related with bleeding in anticoagulated patients with atrial fibrillation 69, 70. We surmised that the discrepancy between our study and previous results was attributed to different cohort. In our study, patients with older age or higher baseline PT level tended to be more careful about their treatment or receive more intensive care from their families, thus the risk for safety events, such as hemorrhinia, was lower. Our study also found that previous anticoagulant use was positively related with bleeding. This suggested that previous anticoagulant use was a bleeding risk factor to be considered in patients who would receive anticoagulant treatment.

Monitoring of hirudin therapy is usually not necessary in patients receiving hirudin prophylactically or without coagulation disturbances 71, 72. However, like all potent anticoagulants, excessive doses of hirudin may cause unwanted bleeding. In patients with predisposition of bleeding, monitoring is necessary to avoid unwanted bleeding. In patients with renal dysfunction, monitoring is also essential because hirudin is mostly eliminated through the kidney, and renal dysfunction may lead to plasma hirudin accumulation 73, 74. Conventional APTT measurement is not suitable for long-term hirudin monitoring because of its small range of linearity with blood levels of hirudin and thus the overdose of hirudin would be easily missed 75, 76. Two suitable hirudin monitoring methods are chromogenic substrate assay and ecarin clotting time (ECT). Both of them are sensitive and are linear over a large concentration range 71, 75. In our study, natural hirudin was administrated as a secondary prevention strategy, and patients with kidney dysfunction and tendency to bleed were excluded. Thus we did not conduct chromogenic substrate assay or ECT test. In addition, the daily dose of a 2.25 g Mauxuekang capsule was previously used in ischemic stroke where no severe bleeding was reported 36, suggesting the safety of such dosage as well. The results from our study indicated that patients taking hirudin combined with aspirin had significantly less bleeding than warfarin. Taken together, Maixuekang capsule could be a safe choice in the secondary prevention of cardioembolic stroke.

In fact, hirudin and its recombinant synthetics have been extensively investigated in both basic and clinical studies. The recombinant hirudin products, such as lepirudin, desirudin and bivalirudin, have manifested their clinical relevance in short term preoperative and postoperative thrombosis prophylaxis 21, disseminated intravascular coagulation 77, coronary ischemic syndromes 25, extracorporeal circulation 78, and heparin-induced thrombocytopenia 79. Currently, bivalirudin, a synthetic hirudin analog comprising 20 amino acid residues from hirudin, shows increasing importance in clinical use for its lower immunogenicity and less dependence on renal clearance 80. Bivalirudin has been approved in the use of percutaneous transluminal coronary angioplasty 81. In recent studies including patients with acute coronary syndrome (ACS) who underwent invasive management, bivalirudin significantly reduced bleeding complications 82 and improved outcomes 25 when compared with unfractionated heparin. However, the efficacy and safety of hirudin and its synthetic in the prophylaxis of cardioembolic stroke have not yet been studied. Our study attempted to test the efficacy and safety of hirudin in the secondary prevention of cardioembolic stroke due to NVAF. With no significant difference in the efficacy and less safety outcome events compared with warfarin, our results suggested that in addition to the above mentioned current utilities of hirudin, hirudin and its analog bear the potential to be used in the intervention of ischemic stroke.

Several limitations in our study should be noted. First, our study had a relatively small sample size and a follow-up of only one year. This may lead to a low incidence of outcome events which may subsequently be a reason for not detecting difference in the efficacy. In this case, the interpretation of the current results should be cautious. It is acceptable to conclude that significant difference was not detected between the two groups, rather than that the two groups had similar efficacy. Future study with larger sample size and longer follow-up would help tackle this problem. Secondly, in order to reflect real world clinical practice, our study was an observational study without randomization. Although we established strict inclusion and exclusion criteria to reduce confounding variables, non-randomization design of our study still may lead to potential confounders. Future randomized clinical trials with more rigorous INR management are promising. In addition, our study only included patients with cardioembolic stroke due to NVAF. Further studies including patients of cardioembolic stroke with various etiology, such as systolic heart failure, recent myocardial infarction, and patent foramen ovale, would provide more information.

In conclusion, in this multicenter prospective cohort study, we investigated the efficacy and safety of hirudin plus aspirin therapy in the secondary prevention of cardioembolic stroke due to NVAF. Significant difference was not detected between natural hirudin plus aspirin therapy and dose-adjusted INR-monitored warfarin treatment in the efficacy to prevent stroke recurrence. The incidence of bleeding was significantly lower in the hirudin plus aspirin group than in the warfarin group. Our study provided preliminary evidence of the efficacy and safety of the hirudin plus aspirin therapy in the secondary prevention of NVAF-induced cardioembolic stroke, and we provided a possible alternative solution to the underuse of warfarin in China. Future RCTs with larger sample size, longer follow-up and more elaborate arm design would be promising.

## Supplementary Material

Supplementary figure S1.

## Figures and Tables

**Table 1:** Baseline characteristics of the hirudin plus aspirin group and the warfarin group

	Hirudin plus Aspirin Group (n=112)	Warfarin Group (n=109)	*P*-value
Age (mean ± SD) (years)	69.94 ± 8.97	68.95 ± 10.17	0.446
Male, n (%)	57 (50.89%)	67 (64.47%)	0.113^a^
GCS score, median (IQR)	12 (10-15)	13 (10-15)	0.822
NIHSS score, median (IQR)	6 (4-10)	6 (4 -9)	0.905
CHA_2_DS_2_-VASc, median (IQR)	5 (4-5)	5 (4-6)	0.708
CHA_2_DS_2_-VASc ≥ 5, n (%)	65 (58.04%)	55 (50.46%)	0.258^a^
HAS-BLED, median (IQR)	3 (2-4)	3 (2-4)	0.646
HAS-BLED ≥ 3, n (%)	75 (66.96%)	68 (62.39%)	0.476^a^
**History, n (%)**			
Hypertension	60 (53.58%)	53 (48.62%)	0.420^a^
Coronary heart disease	51 (45.54%)	40 (36.70%)	0.164^a^
Stroke	35(31.25%)	31 (28.44%)	0.648^a^
Diabetes mellitus	9 (8.04%)	13 (11.93%)	0.334^a^
Hyperlipidemia	13 (11.61%)	17 (15.60%)	0.401^a^
Cigarette smoking	37 (33.04%)	41 (37.61%)	0.476^a^
Drinking	23 (20.54%)	26 (23.85%)	0.553^a^
Previous anticoagulants use, n (%)	34 (30.36%)	39 (35.78%)	0.391^a^
Cholesterol (mean ± SD) (mg/dl)	3.84 ± 1.04	3.75 ± 1.14	0.547
Platelets (mean ± SD) (×10^9^/L)	171.51 ± 42.711	169.14 ± 39.67	0.670
**Coagulation indices**			
PT (mean ± SD) (s)	11.086 ± 0.782	11.102 ±0.789	0.879
TT (mean ± SD) (s)	17.530 ± 2.266	17.346 ± 2.014	0.523
APTT (mean ± SD) (s)	27.593 ± 3.382	27.311 ± 3.738	0.557
INR, median (IQR)	1.08 (0.98-1.22)	1.15 (1.03-1.29)	0.078

Abbreviations: SD = standard deviation; IQR = interquartile range; GCS = Glasgow Coma Scale; NIHSS = National Institute of Health Stroke Scale; CHA_2_DS_2_-VASc = congestive heart failure, hypertension, age ≥ 75 (doubled), diabetes, stroke (doubled), vascular disease, age 65-74 and sex category (female); HAS-BLED = hypertension, abnormal renal/liver function (1 point each), stroke, bleeding history or predisposition, labile international normalized ratio, elderly (>65), drugs/alcohol concomitantly (1 point each); PT = prothrombin time; TT = thrombin time; APTT = activated partial thromboplastin time; INR = international normalized ratio. P values are determined by Student's t-test or Mann-Whitney U test unless designated by^*a*^; p values with^*a*^ are determined by Chi-square test.

**Table 2:** Proportion of patients with different time spent within the INR therapeutic range in warfarin group

Time spent within the INR therapeutic range	Number of patients (%)
0-30%	6 (5.5%)
31-40%	4 (3.7%)
41-50%	14 (12.8%)
51-60%	13 (11.9%)
61-70%	18 (16.5%)
71-80%	54 (49.5%)

Abbreviation: INR = international normalized ratio.

**Table 3:** Clinical outcomes of hirudin plus aspirin group and warfarin group

	Hirudin + Aspirin Group (n=112)	Warfarin Group (n=109)	*χ*^2^* P*-value	Hirudin + Aspirin/Warfirin HR (95%CI)
Total number of efficacy and safety events, n (%)	7 (6.25%)	14 (12.84%)	0.095	N/A
Primary efficacy endpoint				
Recurrence of cardioembolic stroke due to NVAF, n (%)	4 (3.57%)	3 (2.75%)	0.728	1.30 (0.29-5.80)
Composite safety event				
Hemorrhage, death or other serious adverse events, n (%)	3 (2.68%)	11 (10.09%)	0.024	0.27 (0.07-0.95)

Abbreviations: HR = hazard ratio; CI = confidence interval; NVAF = nonvalvular atrial fibrillation.

**Table 4:** Logistic regression analysis for efficacy events within one year after stroke onset

Variables	Univariate analysis			Multivariate analysis		
	OR	95% CI	*P-*value	OR	95% CI	*P-*value
Age (year)	0.987	0.914-1.066	0.743			
Male	4.881	0.578-41.244	0.145			
HA regimen	1.309	0.286-5.988	0.729			
GCS score	1.013	0.793-1.294	0.919			
NIHSS score	0.949	0.789-1.141	0.579			
CHA_2_DS_2_-VASc	0.851	0.463-1.565	0.604			
HAS-BLED	1.140	0.643-2.020	0.654			
**History**						
Hypertension	1.272	0.279-5.822	0.756			
Coronary heart disease	3.692	0.700-19.465	0.124			
Stroke	0.382	0.045-3.237	0.378			
Diabetes mellitus	N/A	N/A	0.998*			
Hyperlipidemia	1.057	0.123-9.105	0.959			
Cigarette smoking	1.390	0.303-6.375	0.672			
Drinking	5.007	1.081-23.186	0.039	5.007	1.081-23.186	0.039
Previous anticoagulant use	0.806	0.153-4.255	0.799			
Cholesterol level	0.789	0.388-1.604	0.513			
Platelets level	0.998	0.980-1.017	0.834			
**Baseline coagulation indices**						
PT	0.876	0.337-2.276	0.785			
TT	1.112	0.784-1.578	0.552			
APTT	1.229	0.965-1.564	0.094			
INR	0.201	0.003-13.294	0.454			

Abbreviations: OR = odds ratio; CI = confidence interval; HA = hirudin plus aspirin; GCS = Glasgow Coma Scale; NIHSS = National Institute of Health Stroke Scale; CHA_2_DS_2_-VASc = congestive heart failure, hypertension, age ≥ 75 (doubled), diabetes, stroke (doubled), vascular disease, age 65-74 and sex category (female); HAS-BLED = hypertension, abnormal renal/liver function (1 point each), stroke, bleeding history or predisposition, labile international normalized ratio , elderly (>65), drugs/alcohol concomitantly (1 point each); PT = prothrombin time; TT = thrombin time; APTT = activated partial thromboplastin time; INR = international normalized ratio. *OR and 95%CI was inapplicable because stroke recurrence (7 cases of stroke recurrence) all happened in patients without Diabetes Mellitus (199 patients without Diabetes Mellitus).

**Table 5:** Logistic regression analysis for composite safety events within one year after stroke onset

Variables	Univariate analysis			Multivariate analysis		
	OR	95% CI	*P*-value	OR	95% CI	*P*-value
Age (year)	0.922	0.873-0.973	0.003	0.897	0.838-0.960	0.002
Male	0.566	0.189-1.689	0.307			
HA regimen	0.245	0.066-0.905	0.035	0.217	0.051-0.925	0.039
GCS score	1.208	0.959-1.520	0.108			
NIHSS score	1.015	0.934-1.103	0.729			
CHA_2_DS_2_-VASc	0.889	0.575-1.375	0.598			
HAS-BLED	0.568	0.361-0.894	0.014			
**History**						
Hypertension	0.943	0.320-2.785	0.916			
Coronary heart disease	0.366	0.099-1.350	0.131			
Stroke	0.168	0.022-1.312	0.089			
Diabetes mellitus	1.558	0.325-7.463	0.579			
Hyperlipidemia	0.469	0.059-3.726	0.474			
Cigarette smoking	1.020	0.329-3.156	0.973			
Drinking	1.440	0.431-4.808	0.553			
Previous anticoagulant use	4.022	1.296-12.478	0.016	6.355	1.690-23.892	0.006
Cholesterol level	0.698	0.415-1.176	0.177			
Platelets level	1.001	0.988-1.015	0.844			
**Baseline coagulation indices**						
PT	0.483	0.236-0.989	0.046	0.328	0.136-0.789	0.013
TT	0.950	0.735-1.228	0.694			
APTT	1.062	0.911-1.239	0.441			
INR	0.970	0.678-1.389	0.869			

Abbreviations: OR = odds ratio; CI = confidence interval; HA = hirudin plus aspirin; GCS = Glasgow Coma Scale; NIHSS = National Institute of Health Stroke Scale; CHA_2_DS_2_-VASc = congestive heart failure, hypertension, age ≥ 75 (doubled), diabetes, stroke (doubled), vascular disease, age 65-74 and sex category (female); HAS-BLED = hypertension, abnormal renal/liver function (1 point each), stroke, bleeding history or predisposition, labile international normalized ratio , elderly (>65), drugs/alcohol concomitantly (1 point each); PT = prothrombin time; TT = thrombin time; APTT = activated partial thromboplastin time; INR = international normalized ratio.

## Abbreviations

NVAF: nonvalvular atrial fibrillation
INR: international normalized ratio
HR: hazard ratio
OR: odds ratio
CI: confidence interval
HA: hirudin plus aspirin
NOACs: new oral anticoagulants
TOAST: Trial of Org 10172 in Acute Stroke Treatment
ICH: intracranial hemorrhage
GCS: Glasgow Coma Scale
NIHSS: National Institute of Health Stroke Scale
CHA2DS2-VASc: congestive heart failure, hypertension, age ≥75 [doubled], diabetes, stroke [doubled], vascular disease, age 65-74, and sex category [female]
HAS-BLED: hypertension, abnormal renal/liver function [1 point each], stroke, bleeding history or predisposition, labile international normalized ratio [INR], elderly [>65], drugs/alcohol concomitantly [1 point each)]
PT: prothrombin time
TT: thrombin time
APTT: activated partial thromboplastin time
ATU: antithrombin units
IU: international unit
TTR: time in therapeutic range
SD: standard deviation
DVT: deep venous thrombosis
ECT: ecarin clotting time
ACS: acute coronary syndrome
RCTs: randomized controlled trials
