# Supplementary material for: The efficacy and safety of Hirudin plus Aspirin versus Warfarin in the secondary prevention of Cardioembolic Stroke due to Nonvalvular Atrial Fibrillation: A multicenter prospective cohort study
Source: Int J Med Sci. 2021 Jan 9;18(5):1167–78. doi: 10.7150/ijms.52752 (PMC7847633; doi:10.7150/ijms.52752)
Supplement: Supplementary file 1 — Supplementary figure S1. [file ijmsv18p1167s1.pdf]

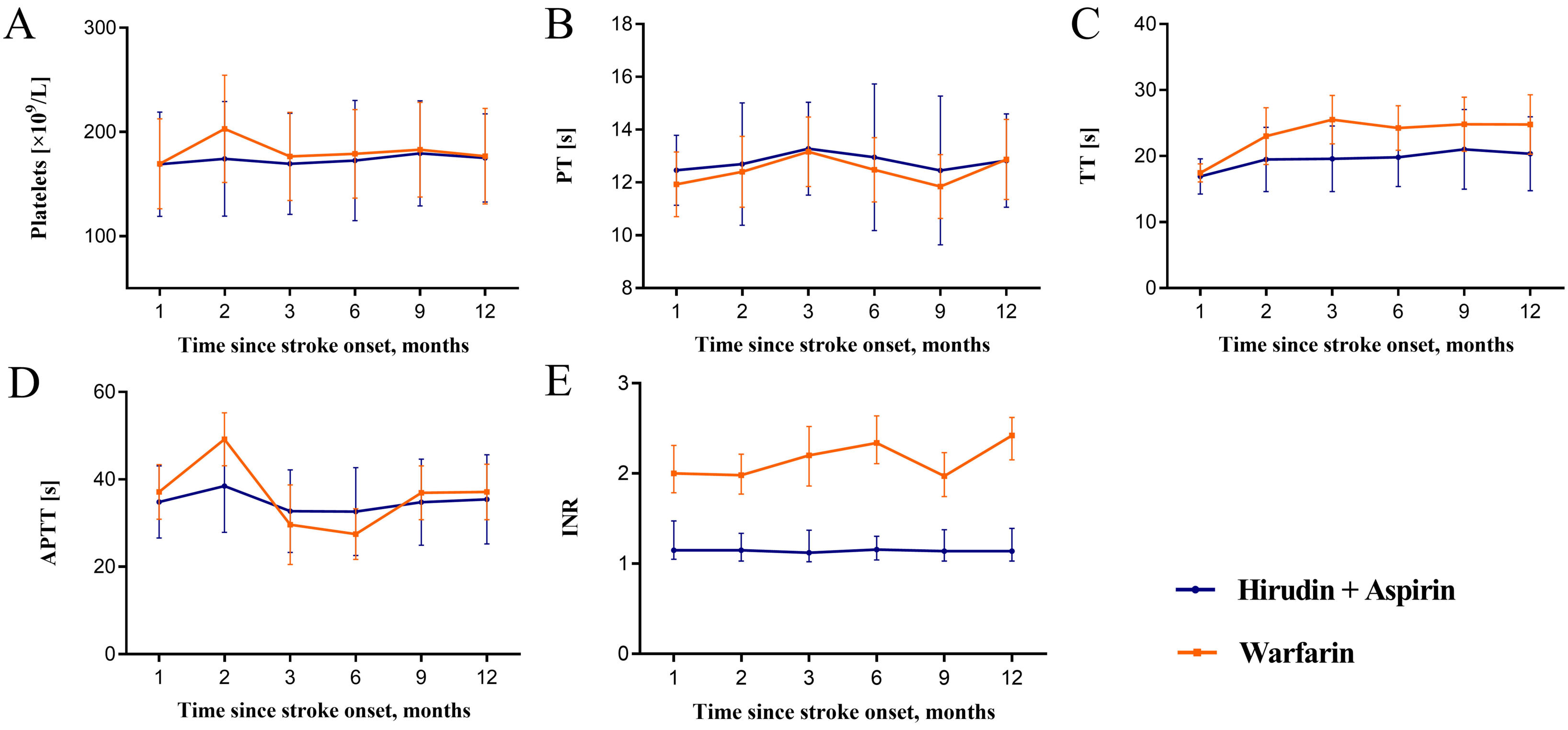

Figure S1. Platelets and coagulation indices during the six follow-ups. Figure (A-E) presents levels of platelets, prothrombin time (PT), thrombin time (TT), activated partial thromboplastin time (APTT), and international normalized ratio (INR), respectively, during the six follow-ups.

Figure (A-D) is presented as mean + standard deviation while Figure (E) is presented as median and interquartile range.
